# Risk factors of newly detected and masked untreated hypertension in a South Asian population of Type-2 diabetes patients

**DOI:** 10.12669/pjms.39.3.7666

**Published:** 2023

**Authors:** Sahrai Saeed, Abukar Mohamed Ali, Muhiddin Abdi Mahmoud, Peter M. Nilsson

**Affiliations:** 1 Sahrai Saeed, MD, PhD. Department of Heart Disease, Haukeland University Hospital, Bergen, Norway; 2 Abukar Mohamed Ali, MD. Department of Heart Disease, Haukeland University Hospital, Bergen, Norway; 3 Muhiddin Abdi Mahmoud, MD. Department of Nephrology, Mnazi Mmoja Referral Hospital, Zanzibar; 4 Peter M. Nilsson, MD, PhD. Department of Clinical Sciences, Lund University, Skane University Hospital, Malmö, Sweden

Hypertension is a well-established cardiovascular risk factor in patients with diabetes, chronic kidney disease, obesity, heart failure and stroke. Among the modifiable risk factors, particularly diabetes together with sedentary lifestyle, smoking and obesity greatly influences cardiovascular risk in patients with hypertension.^1^ In fact, diabetes is the most important cause of chronic kidney disease followed by hypertension. According to the European SCORE (Systematic COronary Risk Evaluation system) risk stratification tool, the presence of diabetes with signs of target organ damage (proteinuria, or with a major risk factor such as Grade-3 hypertension or hypercholesterolaemia) is defined as “very high risk” with a calculated 10-year SCORE risk of ≥10%.

In South Asia, the prevalence of diabetes, hypertension and atherosclerotic cardiovascular disease is on the rise.^2^ Through a comprehensive literature review and our own experiences, we recently demonstrated that South Asian populations, not only living in their native countries, but also in the Western countries, carry a significantly higher risk of diabetes and cardiovascular disease compared with native white Europeans.^2^

In a recent issue of Pakistan Journal of Medical Science (PJMS), Adnan et al.^3^ reported on the prevalence and covariates of hypertension in a study of 129 patients with diabetes (mean age 49 years, 65% females). The prevalence of overall hypertension was 58.1% (n=75) including 43.4% (n=56) with previously known and 14.7% (n=19) newly detected. Among those who had known hypertension, 45 (80.4%) were treated and only 27% in this group had achieved blood pressure (BP) control (clinic BP <140/90 mmHg). Women were more likely to have missed hypertension, while men had greater risk of having untreated hypertension. The definition of hypertension and BP controlled was based upon clinic/office BP with the threshold of 140/90 mmHg.

This is an important study investigating the burden of hypertension and BP control in patients with diabetes and adds useful information to the existing literature. Although the overall prevalence of hypertension was not low in the study, including 24-hour ambulatory BP (ABPM) recording could yield even higher prevalence of true hypertension, including so-called masked hypertension. In fact, 24-hour ABPM measurement not only identifies hypertension subtypes and evaluates treatment response (Fig.1), but can also detect other BP patterns such as blunted nocturnal BP fall (non-dipping) or nocturnal rise, which are not infrequent in patients with diabetes.^4^

**Fig.1:**
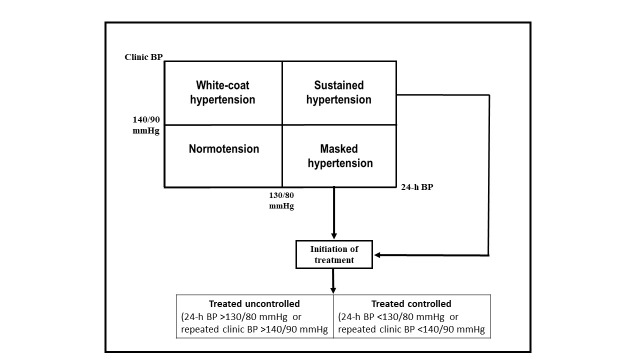
The definition of hypertension by combining office and 24-h blood pressure measurements.

Furthermore, BP control was assessed by office/clinic BP – threshold of 140/90 mmHg. However, for patients with diabetes, chronic kidney disease, and coronary artery disease (excluded by Adnan et al.), which all are defined as high-risk patients, the recommended therapeutic target by most guidelines is towards 135/80 mmHg or 130/80 mmHg in younger patients.

Further, the authors showed that modifiable factors such as lack of education, sedentary lifestyle, and unhealthy diet not only contribute to hypertension, but also increase the risk of undetected hypertension and masked untreated hypertension.

Orthostatic hypotension, which is common in patients with diabetes, was not discussed as it probably was not the aim of the study. However, orthostatic hypotension defined as a reduction in systolic BP of at least 20 mmHg or in diastolic BP of at least 10 mmHg within three minutes of standing, is associated with an increased risk of mortality and cardiovascular events.^5^ Resting heart rate also provides important clinical and prognostic information, both in the general population and in patients with hypertension, why it should also be recorded at the time of BP measurements^6^, which was not the case in the present study.

Finally, although the classes of antihypertensive medications were accounted for, the information about statin use was missing. Patients with hypertension, and Type-2 diabetes often have unfavourable lipid profile (atherogenic dyslipidaemia: elevated triglycerides and LDL cholesterol, and low HDL cholesterol) and may benefit from a statin, which should be added to the patient’s antihypertensive treatment.

## Future perspectives:

Despite the usual prescribed antihypertensive medications with maximal tolerable doses, many patients fail to achieve the recommended BP targets. The novel antidiabetic agents, sodium-glucose cotransporter-2 inhibitors (SGLT2i), can reduce office and ambulatory BP by several mmHg on top of conventional antihypertensive drugs^7^, and may help improve BP control in patients with diabetes, in whom the therapeutic target is often difficult to achieve. Apart from some expert opinions and position papers^8^,^9^, there is very little evidence in the literature on the role of SGLT2i in diabetes as well as in other high-risk patients with cardiovascular disease in Pakistan. This can be the focus of future research.

Diabetes can contribute to subclinical LV dysfunction (diabetic cardiomyopathy) by both the direct effect on the LV myocardium through a load-independent impairment of systolic function, and by arterial stiffening increasing LV afterload.^10^ Future studies from the region should also include novel echocardiographic techniques such as strain imaging derived from speckle-tracking echocardiography for assessment of subclinical LV dysfunction.

Furthermore, Type-2 diabetes is associated with arterial stiffening and abnormal pulsatile arterial hemodynamics in patients with heart failure and preserved ejection fraction.^11^ Hence, assessment of arterial hemodynamics such as arterial stiffness (pulse wave velocity) assessed by applanation tonometry, and systemic arterial compliance are warranted in future well-designed prospective research studies including patients with diabetes.

## CONCLUSIONS

The findings that modifiable factors such as lack of education, sedentary lifestyle and unhealthy diet contribute to hypertension and treatment resistance, as well as increase the risk of undetected hypertension, are important and highlight the need of formal usage, patient education, improving compliance and tackling therapeutic inertia by the physician.

Studies have shown that diabetes is a predictor of resistant hypertension. Other predictors are older age, male gender, black African origin, obesity, established atherosclerotic cardiovascular disease and chronic kidney disease.^12^,^13^ In addition to pharmacological interventions, lifestyle interventions have also been shown to have beneficial effects with regard to reduction in the risk of diabetes by increasing insulin sensitivity, weight loss as well as improved glycaemic and lipid control. Every effort should be made to achieve better glycaemic and BP control in this relatively young population^3^, who are at substantially high risk of future vascular events such as stroke, myocardial infarction and heart failure, as well as cardiovascular mortality.

The opinion expressed in the present expert commentary is the view of the authors and does not necessarily reflect the view of the institutions the authors belong to.

### Author Contributions:

**SS** wrote the first draft of the article which was revised by **AMA, MAM and PMN**.

All authors approved the final submission.
